# Galectin-4 levels in hospitalized versus non-hospitalized subjects with obesity: the Malmö Preventive Project

**DOI:** 10.1186/s12933-022-01559-9

**Published:** 2022-07-02

**Authors:** Johan Korduner, Hannes Holm, Amra Jujic, Olle Melander, Manan Pareek, John Molvin, Lennart Råstam, Ulf Lindblad, Bledar Daka, Margret Leosdottir, Peter M. Nilsson, Erasmus Bachus, Michael H. Olsen, Martin Magnusson

**Affiliations:** 1 Department of Clinical Sciences, Lund University, Jan Waldenströms gata 15, floor 5, Malmö, Sweden; 2 Department of Internal Medicine, Skåne University Hospital, Malmö, Sweden; 3 Department of Cardiology, Skåne University Hospital, Malmö, Sweden; 4 Department of Internal Medicine, Yale New Haven Hospital, Yale School of Medicine, New Haven, CT USA; 5 Department of Cardiology, Copenhagen University Hospital, Gentofte, Denmark; 6 Institute of Medicine, School of Public Health and Community Medicine, Sahlgrenska Academy, University of Gothenburg, Gothenburg, Sweden; 7 Department of Regional Health Research, University of Southern Denmark, Odense, Denmark; 8 Department of Internal Medicine and Steno Diabetes Center Zealand, Holbaek Hospital, Holbaek, Denmark; 9 Wallenberg Center for Molecular Medicine, Lund University, Lund, Sweden; 10 Hypertension in Africa Research Team (HART), North-West University, Potchefstroom, South Africa; 11 Scania University Hospital, 20502 Malmö, Sweden

**Keywords:** Obesity, Cardiovascular disease, Biomarkers, Diabetes, Galectin-4

## Abstract

**Background:**

Obesity is strongly associated with the development of cardiovascular disease (CVD). However, the heterogenous nature of obesity in CVD-risk is still poorly understood. We aimed to explore novel CVD biomarkers and their possible association with presumed unhealthy obesity, defined as hospitalized subjects with obesity (HO).

**Methods:**

Ninety-two proteins associated with CVD were analyzed in 517 (mean age 67 ± 6 years; 33.7% women) individuals with obesity (BMI ≥30 kg/m^2^) from the Malmö Preventive Project cohort, using a proximity extension array technique from the Olink CVD III panel. Individuals with at least one recorded hospitalization for somatic disease prior to study baseline were defined as HO phenotypes. Associations between proteins and HO (n = 407) versus non-hospitalized subjects with obesity (NHO, n = 110), were analyzed using multivariable binary logistic regression, adjusted for traditional risk factors.

**Results:**

Of 92 analyzed unadjusted associations between biomarkers and HO, increased levels of two proteins were significant at a false discovery rate < 0.05: Galectin-4 (Gal-4) and insulin-like growth factor-binding protein 1 (IGFBP-1). When these two proteins were included in logistic regression analyses adjusted for age and sex, Gal-4 remained significant. Gal-4 was independently associated with the HO phenotype in multivariable logistic regression analysis (OR 1.72; CI95% 1.16–2.54). Post-hoc analysis revealed that this association was only present in the subpopulation with diabetes (OR 2.26; CI95% 1.25–4.07). However, an interaction analysis was performed, showing no significant interaction between Gal-4 and prevalent diabetes (p = 0.16).

**Conclusions:**

In middle-aged and older individuals with obesity, increased Gal-4 levels were associated with a higher probability of HO. This association was only significant in subjects with diabetes only, further implying a role for Gal-4 in diabetes and its complications.

**Supplementary Information:**

The online version contains supplementary material available at 10.1186/s12933-022-01559-9.

## Introduction

Obesity (body mass index, BMI ≥30 kg/m^2^) contributes to health complications and reduces life expectancy with up to approximately 20 years [1]. This is mainly due to the significantly increased risk of developing numerous non-communicable diseases, such as type 2 diabetes (DM2), cardiovascular disease (CVD) and certain types of cancer [2, 3]. Even more troublesome, the global prevalence of obesity has been steadily increasing since the 1970s, especially among adolescents and children, today reaching pandemic levels [4]. Even though the link between obesity and increased CVD risk is not a matter of debate per se, there have long been speculations regarding how certain individuals with obesity possess a lower risk of developing CVD and diabetes type 2 (DM2), thus showing a heterogeneity of obesity as a risk factor [5].

Furthermore, although the cardiometabolic complications of obesity are well established from an epidemiological perspective, the underlying pathophysiological mechanisms are not fully understood, particularly when taking into consideration the heterogeneity of obesity [6]. Recently, there have been considerable technological advances in the incorporation of multiomics into exploring alterations in specific cell types and identifying modifications in signaling events that promote disease development [7]. To better understand the mechanisms behind disease progression in obesity, we applied proximity extension assay (PEA) technology to measure 92 proteins (biomarkers) associated with inflammation and CVD [8]. This represents an appealing approach to explore associations between multiple proteins and biological systems, which could in turn present possible diagnostic, prognostic, and therapeutic implications.

The *aim* of this cross-sectional, population-based study was to explore possible novel associations between CVD biomarkers and a phenotype of unhealthy obesity, namely obese subjects with a history of hospitalization for somatic disease up until late mid-life, [9–11] using a multiplex proteomic platform consisting of 92 proteins linked to cardiovascular disease.

## Methods

### Study population

In the 1970s, the Malmö Preventive Project (MPP) cohort was established at the University Hospital, Malmö, Sweden, for the purpose of investigating cardiovascular risk factors in the general population [12]. A total of 33,346 individuals were included at baseline (71% attendance rate, 2/3 men), and survivors of the original cohort were re-examined between 2002 and 2006 (n = 18,240) in the MPP Re-Examination cohort (MPP-RES, attendance rate 72%) [13]. Furthermore, from this MPP-RES cohort, a sub-sample of 1,792 participants was selected to undergo echocardiography and electrocardiogram (ECG) recordings. These individuals were randomly chosen from groups based on their glucometabolic status. Oversampling was performed within the groups with glucometabolic disturbances (impaired fasting glucose, IFG (≥ 6.1 mmol/L or a single measurement of 7.0–11.0 mmol/l of fasting plasma glucose (FPG); new onset diabetes; and prevalent diabetes) to ensure numerical balance, as described previously, [14] resulting in approximately 1/3 normoglycemic subjects, 1/3 with IFG, and 1/3 with diabetes). Prevalent diabetes was defined as either new-onset diabetes (defined by two separate measurements of FPG ≥ 7.0 mmol/l or one measurement ≥ 11.1 mmol/l) or previously known diabetes (obtained through participant self-reporting and/or reporting of current anti-diabetic medication) [14].

**Fig. 1:**
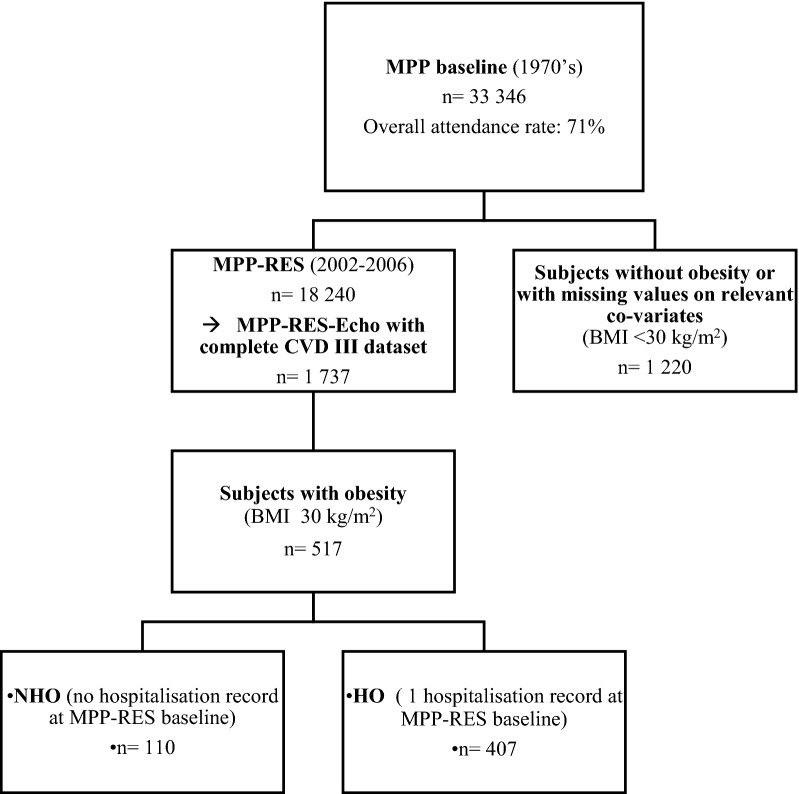
Flow-chart of the MPP-RES cohort stratified for individuals with and without obesity, as well as history of hospitalization for somatic disorders in subjects with obesity, respectively

From the MPP-RES echocardiography sub-cohort, a total of 517 individuals with obesity and complete biomarker data were included in the present study. This subsample was further sub-divided into two different categories based on hospitalization history. Individuals with obesity with at least one recorded history of hospitalization prior to study baseline (n = 407) were defined as hospitalized subjects with obesity (HO). Correspondingly, individuals who had no history of hospitalization for somatic disease up until inclusion at MPP-RES baseline (n = 110) in late mid-age were defined as non-hospitalized subjects with obesity (NHO), (Fig. 1). Data on prior hospitalization was obtained through the Swedish National Hospital Inpatient Register. Normal deliveries were considered non-hospitalization; otherwise, all diagnoses were included. A detailed list of included/excluded diagnoses can be found in Additional file 1: Table S1.

As described in previous publications, [13, 14] data on medical history and lifestyle (including physical activity, alcohol consumption, dietary habits, and smoking status) were acquired through a self-administered questionnaire. Weight (kg) and height (m) were measured in light indoor clothing, and BMI (kg/m^2^) was subsequently calculated. Blood pressure (mmHg) was measured twice using a validated sphygmomanometer with a mercury manometer in the supine position by trained nurses after 10-minutes of rest—the mean values were then recorded. No intra- and/or inter-observed variability calculations were performed; however, the sphygmomanometer used was validated and continuously calibrated according to research standards at Malmö University hospital. Blood samples were acquired after an overnight fast and stored at − 80 °C [15].

### Proteomic profiling

Plasma samples were analyzed by the Proximity Extension Assay (PEA) technique, using the Proseek Multiplex CVD III 96 × 96 reagents kit (Olink Bioscience, Uppsala, Sweden). The technique uses two antibodies that bind pairwise to each specific protein, creating a polymerase chain reaction sequence which then can be detected and quantified. The CVD III panel consists of 92 markers with established or proposed involvement in metabolism, inflammation, or cardiovascular disease (Additional file 2: Table S2). One protein was below the limit of detection in > 15% samples (N-terminal pro-B-type natriuretic peptide, NT-proBNP) and thus excluded; instead, NT-proBNP measurement with an electrochemiluminescence immunoassay was used. The mean intra- and inter-assay variations were 8.1% and 11.4%, respectively. Further information on the assays is available on the Olink homepage (www.olink.com).

### Laboratory analyses

Fasting serum total cholesterol, serum triglycerides, serum high-density lipoprotein and FPG were analyzed using Beckman Coulter LX20 (Beckman Coulter Inc., Brea, USA). Serum low-density lipoprotein concentration (LDL-C) was calculated through Friedewald’s formula [16]. NT-proBNP was measured with an electrochemiluminescence immunoassay (Elecsys; Roche Diagnostics, Basel, Switzerland) at the Department of Clinical Chemistry, Akershus University Hospital, Lorenskog, Norway.

### Statistical analysis

Continuous variables are presented as means (± standard deviation, SD) or medians (25th-75th percentiles). A stratified random sample was created for identification of eligible study subjects. HO and NHO subjects were compared using one-way ANOVA test for normally distributed continuous variables, Mann-Whitney U-test for continuous variables with non-normal distribution, and χ2 test for binary variables. Prior to analysis, skewed variables (FPG) were log-transformed. Unadjusted binary logistic regression models exploring associations between each of the 92 proteins and HO were carried out applying the Benjamini-Hochberg multiple testing correction [17] (false discovery rate, FDR, < 0.05). Significant associations were carried forward to analyses according to *Model 1* (age- and sex-adjusted), and further adjusted according to *Model 2* (total cholesterol, current smoking, hypertension, BMI, prevalent diabetes of any type, and log(FPG)). Hypertension was defined as a measured systolic blood pressure ≥140 mmHg and/or diastolic blood pressure ≥90 mmHg and/or currently on antihypertensive medication. Finally, for associations significant in *Model 2*, a post-hoc analysis was carried out in subjects with and without diabetes using the remaining variables in *Model 2*. Lastly, to test for linearity between remaining variables with significant associations in *Model 2* and independent variables, quartile analyses were carried out. All analyses were carried out using SPSS 25.0 (IBM, Chicago, IL, USA). A nominal two-sided p-value of less than 0.05 was considered statistically significant.

## Results

### Study characteristics

Characteristics of the study population are presented in Table 1. HO individuals were older than NHO. Furthermore, lower levels of total cholesterol and LDL-C, as well as lower systolic and diastolic blood pressures were seen in HO when compared with NHO. However, the use of both lipid- and blood pressure lowering drugs was significantly higher in the HO group. No difference between the two groups were seen in FPG levels, prevalent diabetes, BMI, or waist circumference.

**Table 1:** Characteristics of the study population

	Total	HO	NHO	p
n	517	407	110	
Age (years)	67.2 (± 5.9)	67.7 (± 5.9)	65.4 (± 5.9)	**< 0.001**
Sex (women); n (%)	174 (33.7)	144 (35.4)	30 (27.3)	0.11
BMI (kg/m ^2^ )	33.5 (± 3.3)	33.5(± 3.2)	33.4 (± 3.4)	0.76
Waist (cm)	110.2 (± 10.3)	110.4 (± 10.1)	109.5 (± 10.9)	0.46
Smoker; n (%)	67 (13.0)	54 (13.3)	13 (11.8)	0.67
SBP (mmHg)	149.6 (± 20.4)	148.5 (± 20.1)	153.8 (± 20.9)	**0.01**
DBP (mmHg)	86.1 (± 10.6)	85.6 (± 10.6)	88.0 (± 10.2)	**0.04**
Total cholesterol (mmol/L)	5.3 (± 1.1)	5.2 (± 1.1)	5.6 ± 1.1)	**0.001**
LDL-C (mmol/L)	3.4 (± 1.0)	3.3 (± 1.0)	3.7 (± 0.9)	**0.001**
HDL-C (mmol/L)	1.2 (± 0.3)	1.2 (± 0.3)	1.2 (± 0.4)	0.98
Triglycerides (mmol/L)	1.5 (1.0)	1.5 (1.0)	1.6 (1.2)	0.33
Fasting plasma glucose (mmol/L)	7.4 (± 2.2)	7.4 (± 2.1)	7.2 (2.3)	0.37
Lipid-lowering drugs; n (%)	159 (30.8)	142 (34.9)	17 (15.5)	**< 0.001**
Hypertension; n (%)	467 (90.3)	371 (91.2)	96 (87.3)	0.22
AHT drugs; n (%)	359 (69.4)	302 (74.2)	57 (51.8)	**< 0.001**
Prevalent diabetes; n (%)	262 (50.7)	209 (51.4)	53 (48.2)	0.56

Values are means (± standard deviation), medians (IQR) or numbers (%). *AHT * antihypertensive, *BMI * body mass index, *DBP * diastolic blood pressure, *HDL-C * high density lipoprotein concentration, *HO * hospitalized subjects with obesity, *LDL-C * low density lipoprotein concentration, *NHO * non hospitalized subjects with obesity. Bold values denote statistical significance at the p<0.05

### Biomarker analyses

Of 92 analyzed unadjusted associations between biomarkers and HO, increased levels of two proteins were significant at an FDR < 0.05: Galectin-4 (Gal-4) and insulin-like growth factor-binding protein 1 (IGFBP-1) (Additional file 3: Table S3). When these two proteins were included in logistic regression analyses adjusted for age and sex, Gal-4 remained significant (OR 1.76; CI 95% 1.23–2.51; p = 0.002) whereas IGFBP-1 did not (OR 1.24; CI95% 0.97–1.58; p = 0.087). Each 1 SD increase in Galectin-4 (Gal-4) levels was associated with a higher probability of being HO in the fully adjusted logistic regression model (OR 1.72; CI95% 1.16–2.54; p = 0.007) (Table 2). When further excluding external trauma (n = 38) as a determinant of being HO, the positive association for Gal-4 remained significant (p = 0.024). An interaction analysis was performed, showing no significant interaction between Gal-4 and prevalent diabetes (p = 0.16). However, given the known correlation between these two variables, [18, 19] a post-hoc stratified analysis was carried out and revealed that the association between Gal-4 and HO was only present among patients with diabetes (Table 3). To elucidate if the association between Gal-4 and the probability of being HO was linear, we carried out additional quartile analyses. In *Model 2*, p for trend was 0.009, and further analyses revealed that the risk of being HO was found to be strongest in the upper quartile (Additional file 4: Table S4). Finally, we explored how diabetes prevalence and glucose levels differed across quartiles of Gal-4 levels. The highest proportion of subjects with diabetes was found in the upper quartile (Q4) of Gal-4 (65.9%), compared to 27.9% in the lowest quartile of Gal-4 (p for difference between groups = 9.6 × 10^− 9^). Similarly, glucose levels were higher in the upper quartile (Q4) of Gal-4 (p for difference between Q1 and Q4 = 6.1 × 10^− 7^) as compared with Q1.

**Table 2:** Logistic regression models displaying associations of Galectin-4 levels and probability of being HO

	HO (n = 407) vs. NHO (n = 110)	
	OR (CI95%)	p
Unadjusted		
Galectin-4	2.03 (1.42–2.90)	**< 0.001**
Model 1		
Galectin-4	1.85 (1.28–2.67)	**0.001**
Age	1.05 (1.01–1.09)	**0.013**
Sex	0.93 (0.56–1.53)	0.765
Model 2		
Galectin-4	1.72 (1.16–2.54)	**0.007**
Age	1.05 (1.00–1.09)	**0.030**
Sex	0.73 (0.42–1.25)	0.246
Diabetes	0.60 (0.33–1.10)	0.098
Total cholesterol	0.71 (0.56–0.86)	**< 0.001**
Smoking	1.34 (0.67–2.65)	0.407
Hypertension	1.03 (0.50–2.11)	0.938
BMI	1.00 (0.93–1.07)	0.885
FPG	1.27 (0.92–1.75)	0.140

Values are odds ratios (OR) and 95% confidence intervals. Bold values denote statistical significance at the p<0.05

*BMI* body mass index, *FPG* fasting plasma glucose, *HO* hospitalized subjects with obesity

.

**Table 3:** Post-hoc analysis comparing levels of Gal-4 in obese subjects with or without prevalent diabetes

	Subjects without diabetes		Subjects with diabetes	
	n = 255		n = 262	
	HO n = 198; NHO n = 57		HO n = 209; NHO n = 53	
Model 1	OR (CI95%)	p	OR (CI95%)	p
Galectin-4	1.52 (0.99–2.53)	0.111	2.45 (1.38–4.35)	**0.002**
Age	1.06 (1.01–1.12)	**0.024**	1.07 (1.01–1.13)	**0.016**
Sex	1.08 (0.56–2.09)	0.824	0.62 (0.29–1.35)	0.228

Model 2	OR (CI95%)	p	OR (CI95%)	p
Galectin-4	1.45 (0.84–2.49)	0.172	2.26 (1.25–4.07)	**0.007**
Age	1.06 (1.00-1.12)	**0.039**	1.03 (0.97–1.10)	0.279
Sex	0.93 (0.44–1.96)	0.843	0.41 (0.18–0.97)	**0.043**
Total choles§terol	0.92 (0.67–1.25)	0.574	0.60 (0.44–0.81)	**0.001**
Current smoker	1.97 (0.74–5.29)	0.177	1.01 (0.38–2.68)	0.991
Hypertension	1.09 (0.46–2.57)	0.849	0.84 (0.23–3.03)	0.784
BMI	1.08 (0.95–1.23)	0.250	0.94 (0.86–1.03)	0.200
FPG	1.89 (0.88–3.99)	0.063	1.15 (0.80–1.63)	0.454

Values are odds ratios (OR) and 95% confidence intervals. Bold values denote statistical significance at the p<0.05

*BMI* body mass index, *FPG* fasting plasma glucose, *HO* hospitalized subjects with obesity,* NHO* non hospitalized subjects with obesity

## Discussion

By using a newly adopted definition of metabolic health in obesity, based on history of hospitalization for somatic disorders up until late mid-life, [9–11, 20] we found that increased levels of Gal-4 were independently associated with a higher probability of having been hospitalized in a cohort of middle-aged and older obese subjects. Descriptive data at baseline examination did not reveal any differences in neither BMI nor waist circumference between HO and NHO, suggesting a similar fat distribution. However, plasma total cholesterol, LDL-C and blood pressure were significantly lower among HO, likely because of a higher prevalence of medical treatment with both anti-hypertensive and lipid-lowering drugs. Finally, the positive association between Gal-4 and the HO phenotype was significant only in subjects with diabetes.

We have previously carried out cross-sectional studies in the Malmö Diet and Cancer Study cohort, where NHO was defined by using a novel approach of a history of non-hospitalization for somatic disorders up until mid-life [9–11]. In those studies we found that NHO had a decreased risk of both total mortality and incident CVD compared with HO during a 20-year follow-up period. When comparing NHO with non-obese controls, there were no significant differences in terms of mortality or CVD risk [9]. Potential protective factors included a more favorable lipid and glucose profile, downregulation of potentially harmful proteomic biomarkers and a less sedentary lifestyle [10]. Moreover, lower plasma levels of antibodies against anti-phosphorylcholine, which possess anti-inflammatory properties and is coupled with lower CVD risk, were associated with a higher risk of being HO [11]. This is in line with previous research focusing on obesity phenotypes with different cardiometabolic disease risk but with a different terminology, namely metabolically healthy obesity (MHO) [20, 21].

### Metabolically healthy obesity (MHO)

The evolving concept of MHO describes obese individuals that through proposed protective mechanisms, such as peripheral body fat distribution, lower grade of chronic inflammation and higher insulin sensitivity, seem to escape metabolic or cardiovascular complications [20–22]. This description could be considered controversial, since increasing evidence suggests that MHO is not a steady state and can transform into metabolically unhealthy obesity over time. Moreover, when compared with metabolically healthy individuals with normal weight, there is a significantly increased risk for incident CVD and metabolic complications linked to MHO [23–26]. One major concern about the conflicting results lies in the definition of MHO which differs substantially between different studies, but mainly focuses on the absence of risk variables included in the metabolic syndrome [27]. There is now an ongoing debate as to whether the term MHO should be avoided and instead be treated as a conceptual model to study mechanisms linking obesity to risk for or protection from cardiometabolic complications [28].

### Galectin-4

Being part of the galectin family (consisting of 15 small leptin peptides), Gal-4 is expressed almost exclusively in the gastrointestinal tract of healthy individuals, where it plays a role in controlling intestinal inflammation. It reduces proinflammatory cytokine production in the intestinal mucosa, and knockdown of the Gal-4 peptide promotes colorectal cancerogenesis. This suggests that Gal-4 plays a significant role in the pathophysiology of the development of both inflammatory bowel disease and colorectal cancers [29]. However, the physiological role of Gal-4 is multifaceted and further include apical protein trafficking, lipid raft stabilization, intestinal wound healing and bacterial pathogen fighting [30]. Epidemiological data also strongly propose an involvement of Gal-4 in cardiometabolic diseases, suggesting it may be considered as a predictive biomarker for the development of CVD and diabetes [18]. Still, the causal pathway is poorly understood [13, 19]. One theory might lie at the cellular level, where Gal-4 is part of the apical protein transport from the Golgi-apparatus to the apical cell membrane of the enterocyte, including the well-known protease dipeptidyl peptidase-4 (DPP-4) [31]. In mice, DPP-4 seems to be misguided and accumulates intracellularly when Gal-4 is depleted [31]. DPP-4 plays a major role in promoting cardiometabolic disease by cleaving and thus inactivating glucose-dependent insulinotropic polypeptide and glucagon-like peptide 1 (GLP-1), i.e., two of our most common incretins [32]. Modern anti-diabetic drugs such as DPP4-inhibitors and GLP-1 agonists are incretin-based and part of the standard treatment of DM2 as second-line drugs in most patients [33]. Incretins are involved in appetite control and delaying gastric emptying actions that are dependent on GLP-1 receptor activation within the central nervous system, thus having the potential to regulate body weight [34]. Furthermore, another study of women with gestational diabetes found an overexpression of Gal-4 in the placental syncytiotrophoblast cells, compared to healthy controls [35]. Thus, one proposed explanation for our main finding may be Gal-4’s involvement in the development of diabetes, which also has been suggested in a previous publication with a similar approach of proteomic exploration [36]. To elucidate this, we carried out a post-hoc analysis, suggesting that elevation in Gal-4 was associated with higher probability of being HO only in those with prevalent diabetes.

Gal-4 has a potential inflammatory role in the intestinal mucosa. Previous studies have linked obesity and diabetes to altered composition of the gut microbiota [37, 38]. Changes in gut microbiota, i.e., through an unhealthy diet, lead to damage of the intestinal barrier, promote leakage and thus endotoxemia through higher levels of lipopolysaccharides systemically, which in turn stimulates the development of low-grade systemic inflammation associated with the negative impact of both obesity and metabolic disorders [37]. Therefore, Gal-4 might, at least in theory, aggravate the pathological processes induced by the obese-diabetic microbiota.

### Study strengths and limitations

By using a definition of individuals with obesity with a more favorable metabolic health as not having been hospitalized for somatic disease up until late midlife, we were able get an objectively defined and more stable phenotype which could serve as an alternative to the conventional way of defining metabolic health within the population with obesity, commonly called MHO. Previous definitions focus on the absence of criteria for the metabolic syndrome, which could shift intra-individually during repeated measurements at different occasions. Moreover, by renaming metabolic health in obesity as non-hospitalized versus hospitalized individuals with obesity instead of MHO, we avoid the perception of certain phenotypes of obesity labeled as healthy.

There are limitations to this study. Its cross-sectional nature precludes any conclusions about causality. However, the study subjects come from a well-characterized, retrospective cohort with excellent national, and well-validated, register data on hospitalization, which is why it was possible to apply our approach to define NHO and HO. This study only covers individual data collected at one regional center. A multicenter study to replicate the findings would be preferable, but to reduce false positive findings, the use of FDR analysis was carried out. Furthermore, because our subjects were of European descent, these findings might not be generalizable to other populations. Similarly, the population selection based on glucometabolic disturbances could raise concerns of how well this cohort represents the general population. However, when compared with similar cohorts, the incidence rate of diabetes was proportionate [39, 40]. The Olink CVD III panel is partially restricted to proteins associated with CVD and inflammation, and an extended analysis including biomarkers related to diabetes and/or metabolism would most likely add information about the pathophysiology in HO. Lastly, another limitation of this study was that subjects with a non-hospitalization status prior to baseline could still suffer from cardiometabolic disturbances, since no pre-defined diagnoses of hospitalization were decided upon, and many individuals could be treated for chronic illnesses within a primary health care unit. On the other hand, these conditions could have been milder or counterbalanced by protective mechanisms in the affected subjects leading to a status of non-hospitalization in our analyses.

## Conclusions

In obese subjects during late mid-life, increased Galectin-4 levels were associated with a higher probability of being an individual with a history of HO. This association was only significant in subjects with diabetes, implying a role for Galectin-4 in diabetes and its complications.

## Supplementary Information

**Additional file 1: Table S1**. List of all causes of hospitalization in the HO subgroup

**Additional file 2: Table S2**. List of all 92 proteins included in analyses.

**Additional file 3: Table S3**. False discovery rate (FDR) detection of all 92 proteins included in the analyses.

**Additional file 4: Table S4**. Quartile analyses of the association between Gal-4 and the probability of being HO.

## Data Availability

The datasets used and/or analysed during the current study are available from the corresponding author on reasonable request.

## Abbreviations

BMI: Body mass index
CVD: Cardiovascular disease
DM2: Type-2 diabetes
Gal-4: Galectin-4
GLP-1: Glucagon-like peptide 1
HO: Hospitalized subjects with obesity
IFG: Impaired fasting glucose
MHO: Metabolically healthy obesity
MPP: Malmö Preventive Project
MPP-RES: MPP-Re-Examination Study
MUO: Metabolically unhealthy obesity
NHO: Non-hospitalized subject with obesity
NOC: Non-obese controls
NT-proBNP: N-terminal pro-b-type natriuretic peptide
SD: Standard deviation
