# Supplementary material for: Galectin-4 levels in hospitalized versus non-hospitalized subjects with obesity: the Malmö Preventive Project
Source: Cardiovasc Diabetol. 2022 Jul 2;21:125. doi: 10.1186/s12933-022-01559-9 (PMC9250274; doi:10.1186/s12933-022-01559-9)
Supplement: Supplementary file 1 — Additional file 1: Table S1. List of all causes of hospitalization in the HO subgroup [file 12933_2022_1559_MOESM1_ESM.docx]

**Supplementary Table S1**

**List of all causes of hospitalization in the HO subgroup.**

| **Cause of hospitalization** | **n** |
| --- | --- |
| Infections | 27 |
| Malignant tumours | 22 |
| Benign tumours | 16 |
| Unidentified tumour | 2 |
| Diabetes | 7 |
| Other endocrine disorder/disease | 5 |
| Pathologic personality disorder | 1 |
| Alcoholism | 6 |
| Drug abuse | 1 |
| Neurological disorder/disease | 5 |
| Oftalmological disorder/disease | 6 |
| Otorinolaryngological disorder/disease | 7 |
| Cardiovascular disorder/disease | 62 |
| Pulmonary disorder/disease | 17 |
| Gastrointestinal disorder/disease | 64 |
| Urogenital disorder/disease | 41 |
| Dermatological disorder/disease | 3 |
| Musculoskeletal disorder/diseases | 37 |
| Congenital malformations | 5 |
| Symptoms without diagnosis | 35 |
| External trauma | 38 |
| **TOTAL** | 407 |
