# Supplementary material for: Galectin-4 levels in hospitalized versus non-hospitalized subjects with obesity: the Malmö Preventive Project
Source: Cardiovasc Diabetol. 2022 Jul 2;21:125. doi: 10.1186/s12933-022-01559-9 (PMC9250274; doi:10.1186/s12933-022-01559-9)
Supplement: Supplementary file 2 — Additional file 2: Table S2. List of all 92 proteins included in analyses. [file 12933_2022_1559_MOESM2_ESM.docx]

**Supplementary Table S2.**

**List of all 92 proteins included in analyses.**

| Aminopeptidase N (AP-N) |
| --- |
| Azurocidin (AZU1) |
| Bleomycin hydrolase (BLM hydrolase) |
| C-C motif chemokine 15 (CCL15) |
| C-C motif chemokine 16 (CCL16) |
| C-C motif chemokine 24 (CCL24) |
| C-X-C motif chemokine 16 (CXCL16) |
| Cadherin-5 (CDH5) |
| Carboxypeptidase A1 (CPA1) |
| Carboxypeptidase B (CPB1) |
| Caspase-3 (CASP-3) |
| Cathepsin D (CTSD) |
| Cathepsin Z (CTSZ) |
| CD166 antigen (ALCAM) |
| Chitinase-3-like protein 1 (CHI3L1) |
| Chitotriosidase-1 (CHIT1) |
| Collagen alpha-1(I) chain (COL1A1) |
| Complement component C1q receptor (CD93) |
| Contactin-1 (CNTN1) |
| Cystatin-B (CSTB) |
| E-selectin (SELE) |
| Elafin (PI3) |
| Ephrin type-B receptor 4 (EPHB4) |
| Epidermal growth factor receptor (EGFR) |
| Epithelial cell adhesion molecule (Ep-CAM) |
| Fatty acid-binding protein, adipocyte (FABP4) |
| Galectin-3 (Gal-3) |
| Galectin-4 (Gal-4) |
| Granulins (GRN) |
| Growth/differentiation factor 15 (GDF-15) |
| Insulin-like growth factor-binding protein 1 (IGFBP-1) |
| Insulin-like growth factor-binding protein 2 (IGFBP-2) |
| Insulin-like growth factor-binding protein 7 (IGFBP-7) |
| Integrin beta-2 (ITGB2) |
| Intercellular adhesion molecule 2 (ICAM-2) |
| Interleukin-1 receptor type 1 (IL-1RT1) |
| Interleukin-1 receptor type 2 (IL-1RT2) |
| Interleukin-17 receptor A (IL-17RA) |
| Interleukin-18-binding protein (IL-18BP) |
| Interleukin-2 receptor subunit alpha (IL2-RA) |
| Interleukin-6 receptor subunit alpha (IL-6RA) |
| Junctional adhesion molecule A (JAM-A) |
| Kallikrein-6 (KLK6) |
| Low-density lipoprotein receptor (LDL receptor) |
| Lymphotoxin-beta receptor (LTBR) |
| Matrix extracellular phosphoglycoprotein (MEPE) |
| Matrix metalloproteinase-2 (MMP-2) |
| Matrix metalloproteinase-3 (MMP-3) |
| Matrix metalloproteinase-9 (MMP-9) |
| Metalloproteinase inhibitor 4 (TIMP4) |
| Monocyte chemotactic protein 1 (MCP-1) |
| Myeloblastin (PRTN3) |
| Myeloperoxidase (MPO) |
| Myoglobin (MB) |
| N-terminal prohormone brain natriuretic peptide (NT-proBNP) |
| Neurogenic locus notch homolog protein 3 (Notch 3) |
| Osteopontin (OPN) |
| Osteoprotegerin (OPG) |
| P-selectin (SELP) |
| Paraoxonase (PON3) |
| Peptidoglycan recognition protein 1 (PGLYRP1) |
| Perlecan (PLC) |
| Plasminogen activator inhibitor 1 (PAI) |
| Platelet endothelial cell adhesion molecule (PECAM-1) |
| Platelet glycoprotein VI (GP6) |
| Platelet-derived growth factor subunit A (PDGF subunit A) |
| Proprotein convertase subtilisin/kexin type 9 (PCSK9) |
| Protein delta homolog 1 (DLK-1) |
| Pulmonary surfactant-associated protein D (PSP-D) |
| Resistin (RETN) |
| Retinoic acid receptor responder protein 2 (RARRES2) |
| Scavenger receptor cysteine-rich type 1 protein M130 (CD163) |
| Secretoglobin family 3A member 2 (SCGB3A2) |
| Spondin-1 (SPON1) |
| ST2 protein (ST2) |
| Tartrate-resistant acid phosphatase type 5 (TR-AP) |
| Tissue factor pathway inhibitor (TFPI) |
| Tissue-type plasminogen activator (t-PA) |
| Transferrin receptor protein 1 (TR) |
| Trefoil factor 3 (TFF3) |
| Trem-like transcript 2 protein (TLT-2) |
| Tumor necrosis factor ligand superfamily member 13B (TNFSF13B) |
| Tumor necrosis factor receptor 1 (TNF-R1) |
| Tumor necrosis factor receptor 2 (TNF-R2) |
| Tumor necrosis factor receptor superfamily member 10C (TNFRSF10C) |
| Tumor necrosis factor receptor superfamily member 14 (TNFRSF14) |
| Tumor necrosis factor receptor superfamily member 6 (FAS) |
| Tyrosine-protein kinase receptor UFO (AXL) |
| Tyrosine-protein phosphatase non-receptor type substrate 1 (SHPS-1) |
| Urokinase plasminogen activator surface receptor (U-PAR) |
| Urokinase-type plasminogen activator (uPA) |
| von Willebrand factor (vWF) |
