# Supplementary material for: Galectin-4 levels in hospitalized versus non-hospitalized subjects with obesity: the Malmö Preventive Project
Source: Cardiovasc Diabetol. 2022 Jul 2;21:125. doi: 10.1186/s12933-022-01559-9 (PMC9250274; doi:10.1186/s12933-022-01559-9)
Supplement: Supplementary file 3 — Additional file 3: Table S3. False discovery rate (FDR) detection of all 92 proteins included in the analyses. [file 12933_2022_1559_MOESM3_ESM.docx]

**Supplementary Table S3.**

**False discovery rate (FDR) detection of all 92 proteins included in the analyses.**

| **Variable** | **p** | **FDR-adjusted p** |
| --- | --- | --- |
| PAI | 0.255 | 0.639 |
| Ep-CAM | 0.239 | 0.639 |
| CHI3L1 | 0.107 | 0.485 |
| ST2 | 0.684 | 0.885 |
| TNFRSF14 | 0.140 | 0.485 |
| LDL-receptor | 0.536 | 0.808 |
| ITGB2 | 0.803 | 0.936 |
| IL17RA | 0.661 | 0.870 |
| TNFR2 | 0.148 | 0.485 |
| MMP9 | 0.277 | 0.661 |
| EPHB4 | 0.370 | 0.756 |
| IL2-RA | 0.280 | 0.661 |
| OPG | 0.451 | 0.798 |
| ALCAM | 0.257 | 0.639 |
| TFF3 | 0.010 | 0.131 |
| SELP | 0.855 | 0.936 |
| CSTB | 0.025 | 0.256 |
| MCP1 | 0.432 | 0.798 |
| CD163 | 0.443 | 0.798 |
| Gal-3 | 0.008 | 0.123 |
| GRN | 0.153 | 0.485 |
| MEPE | 0.532 | 0.808 |
| BLM-hydrolase | 0.826 | 0.936 |
| PLC | 0.072 | 0.473 |
| LTBR | 0.908 | 0.956 |
| Notch-3 | 0.446 | 0.798 |
| TIMP4 | 0.144 | 0.485 |
| CNTN1 | 0.837 | 0.936 |
| CDH5 | 0.579 | 0.834 |
| TLT-2 | 0.849 | 0.936 |
| FABP4 | 0.080 | 0.485 |
| TFPI | 0.492 | 0.808 |
| CCL24 | 0.250 | 0.639 |
| TR | 0.007 | 0.123 |
| TNFRSF10C | 0.351 | 0.734 |
| GDF-15 | 0.005 | 0.115 |
| SELE | 0.192 | 0.552 |
| AZU1 | 0.085 | 0.485 |
| DLK-1 | 0.292 | 0.664 |
| SPON1 | 0.296 | 0.664 |
| MPO | 0.110 | 0.485 |
| CXCL16 | 0.179 | 0.531 |
| IL-6RA | 0.574 | 0.834 |
| RETN | 0.036 | 0.326 |
| **IGFBP-1** | **0.001** | **0.046** |
| CHIT1 | 0.039 | 0.326 |
| TRAP | 0.096 | 0.485 |
| CCL22 | 0.131 | 0.485 |
| PSP-D | 0.894 | 0.956 |
| PI3 | 0.333 | 0.712 |
| AP-N | 0.959 | 0.980 |
| AXL | 0.850 | 0.936 |
| IL-1RT1 | 0.700 | 0.885 |
| MMP2 | 0.662 | 0.870 |
| FAS | 0.790 | 0.936 |
| MB | 0.491 | 0.808 |
| TNFSF13B | 0.133 | 0.485 |
| PRTN3 | 0.166 | 0.509 |
| PCSK9 | 0.123 | 0.485 |
| U-PAR | 0.061 | 0.467 |
| OPN | 0.517 | 0.808 |
| CTSD | 0.832 | 0.936 |
| PGLYRP1 | 0.477 | 0.808 |
| CPA1 | 0.950 | 0.980 |
| JAM-A | 0.607 | 0.842 |
| **Gal-4** | **0.000176** | **0.016** |
| IL-1RT2 | 0.303 | 0.664 |
| SHPS-1 | 0.523 | 0.808 |
| CASP-3 | 0.407 | 0.797 |
| uPA | 0.980 | 0.980 |
| CPB1 | 0.632 | 0.855 |
| tPA | 0.741 | 0.921 |
| SCGB3A2 | 0.128 | 0.485 |
| EGFR | 0.613 | 0.842 |
| IGFBP-7 | 0.580 | 0.834 |
| CD93 | 0.478 | 0.808 |
| IL-18BP | 0.416 | 0.797 |
| COL1A1 | 0.507 | 0.808 |
| PON3 | 0.151 | 0.485 |
| CTSZ | 0.797 | 0.936 |
| MMP-3 | 0.969 | 0.980 |
| RARRES2 | 0.239 | 0.639 |
| ICAM-2 | 0.894 | 0.956 |
| KLK6 | 0.702 | 0.885 |
| PDGF_subunit_A | 0.914 | 0.956 |
| TNF-R1 | 0.066 | 0.467 |
| IGFBP-2 | 0.106 | 0.485 |
| vWF | 0.787 | 0.936 |
| PECAM-1 | 0.379 | 0.758 |
| CCL16 | 0.613 | 0.842 |
| CCL15 | 0.002 | 0.061 |
| NT-proBNP | 0.015 | 0.173 |
