# Supplementary material for: Galectin-4 levels in hospitalized versus non-hospitalized subjects with obesity: the Malmö Preventive Project
Source: Cardiovasc Diabetol. 2022 Jul 2;21:125. doi: 10.1186/s12933-022-01559-9 (PMC9250274; doi:10.1186/s12933-022-01559-9)
Supplement: Supplementary file 4 — Additional file 4: Table S4. Quartile analyses of the association between Gal-4 and the probability of being HO. [file 12933_2022_1559_MOESM4_ESM.docx]

**Supplementary table S4**

**Quartile analyses of the association between Gal-4 and the probability of being HO.**

| **Gal-4 levels** | **OR (CI95%)** | **p-value** |
| --- | --- | --- |
|  |  |  |
| **First quartile** | 1.0 (referent) | - |
| **Second quartile** | 1.14 (0.63-2-06) | 0.66 |
| **Third quartile** | 1.13 (0.63-2.05) | 0.68 |
| **Fourth quartile** | 3.21 (1.48-6.94) | 0.003 |

Values are odds ratios (OR) and 95% confidence intervals for the probability of being HO from logistic regression analyses (n= 407). The first quartile represents the lowest values of Gal-4 whereas the fourth quartile represents the highest values of Gal-4. Regressions are adjusted for age, sex, diabetes, total cholesterol, current smoker, hypertension, body mass index and fasting plasma glucose.
